# Emergency medicine residency fact board: Why our attempt to encourage on-shift learning failed

**DOI:** 10.1007/s40037-019-0508-3

**Published:** 2019-04-05

**Authors:** Kimberly Sokol, Alisa Wray, Megan Boysen-Osborn, Warren Wiechmann, Kathryn Bennett, Shannon Toohey

**Affiliations:** 1 Department of Emergency Medicine, Kaweah Delta Medical Center, Visalia, USA; 2 Department of Emergency Medicine, University of California, Irvine, USA

**Keywords:** residency education, emergency medicine, technology in learning

## Abstract

Residents commonly feel that a lack of time is a significant barrier in keeping up-to-date with both medical knowledge and literature. In this study, we addressed that barrier by placing an iPad-based live fact feed in the resident workroom of our emergency department, therefore allowing for passive learning while on shift. We hypothesized that residents with access to the live feed would score higher on monthly post-curriculum block exams. We ended up finding that the residents actually prefer a more active approach to learning and that many more people in the emergency department other than the residents benefitted from the fact board.

## The story

A large survey of almost 500 emergency medicine residents performed over the past 12 years demonstrated that their lack of time has been a significant barrier in keeping up-to-date with both medical knowledge and literature, hindering their overall competency at work[1]. Residency programs in all fields of medicine have implemented innovative strategies that involve conferences, didactics, online learning modules, practice testing, and other forms of asynchronous learning in efforts to formulate ideal models for academic instruction and learning. Standardized exam preparation courses and other classroom-based curricula have been adopted by residents across the nation to review topics covered in their curriculum while off duty. However, all of these modules require additional time outside of work for the residents to work through. Our residency program wanted to trial an engaging educational intervention that could be delivered on-demand during resident work hours.

Acting on the wake of ample literature [2–4] showing resident satisfaction from use of electronic materials when studying for their annual in-training exam, our emergency medicine residency program implemented an iPad™ based fact board allowing for passive knowledge by residents throughout their shifts. By using a live, rotating feed of slides with facts pertaining to emergency medicine displayed on an iPad™ in a common work area devoted to residents in the department, residents could take multiple short blocks of time throughout their shift to learn quick facts and study for a post-curriculum block exam without taking time away from patient care or requiring additional time outside of work.

Our residency program uses a systems-based curriculum, with 13 different topics per year. At the end of each systems-based block, each resident took a quiz with questions based on the system covered for that block, regardless of whether they had been on an emergency medicine rotation for that block. The fact board contained a mix of 100 facts and questions pertaining to that same system and would change with each block. At the end of the 2014–2015 academic year, the academic year when the fact board was used, we analyzed the quiz scores of the residents who had been in the department, and therefore had access to the fact board, compared with the scores of the residents on off-service rotations, who did not have access to the fact board. We hypothesized that the implementation of the fact board would result in improved examination scores for monthly post-curriculum block examinations for the residents who had access to the fact board when compared with those who did not.

## Surprising outcomes

While there was significant interest in the fact board after it was initially implemented, and residents would frequently look at the fact board and discuss the questions and facts provided, we found that after several months the novelty of the board wore off and it was largely ignored. Our study showed no statistically significant increase in exam scores of those residents who had access to the fact board in the department compared with those without access. However, we also sent a web-based survey to each resident who had participated in the study to elicit their opinions on the fact board, and ended up learning some surprising information.

While we had initially intended this experiment to be a more passive learning experience for the residents, where they could look up at the board at any given time throughout their shift to learn a new fact, multiple residents commented in a free response section of the survey that they would have preferred a more active use of the board, where it could be picked up and used in a format resembling flashcard repetition. One resident suggested having residents stop on the hour to review a ‘question of the hour’, and multiple other residents advocated for integration of the fact board feed for senior resident and attending teaching moments. This year we will be implementing a fact board that is changed manually and can be used for attending physician teaching in a ‘fact per day’ format, while also allowing for the fact board to be picked up by the residents at any point throughout their shift to teach themselves or quiz others.

Another even more surprising outcome was how much the fact board brought together the residents with other members of the hospital, such as emergency department ancillary staff and consultants from other specialties. Nurses and technicians would frequently stand in front of the board and quiz each other on facts, then ask the residents for clarification on the answer to the question. Consultants would come to the emergency department to see a patient, then stop in the resident work room to read a few facts and even use it to teach their fellows, residents, and medical students if the topic happened to pertain to their patient. One of our nursing supervisors adopted the fact board concept to use in the nursing department to display various learning points and public service announcements for the nurses after seeing their satisfaction with the board.

## Lessons learned

When designing our project and study, we knew that residents’ primary complaint about learning new material was related to the lack of available time. This resulted in a solution that was too focused on passive learning and as such was not very effective and was ultimately ignored after the initial novelty wore off. While the idea of passive learning sounds good in theory, realistically it rarely is effective.

The experience was a reminder that residents still want to be taught, but they want to be taught in a way that maximizes their time. Utilizing down time during shifts for teaching moments, random pearls and test review can be a valuable teaching tool, if done properly. The idea of having a fact board with a variety of teaching pearls available as a cue for active teaching moments has merits, but still needs to be used actively by teachers so that learners can benefit from them.

## Moral of the story

The initial failure of utilizing a residency fact board on shift reminded us that residents appreciate active learning while on shift, and anything that is not actively used will eventually fade into the background and become forgotten. Additionally, we were reminded the learning does not stop with the residents—nurses, technicians, and consultants from other specialties all found the fact board interesting, specifically when they used it in an active manner to start a discussion with other residents or staff.
